# Prediction and quality assessment of protein quaternary structure models using the MultiFOLD2 and ModFOLDdock2 servers

**DOI:** 10.1093/nar/gkaf336

**Published:** 2025-04-25

**Authors:** Liam J McGuffin, Shaima N Alhaddad, Behnosh Behzadi, Nicholas S Edmunds, Ahmet G Genc, Recep Adiyaman

**Affiliations:** School of Biological Sciences, University of Reading, Whiteknights, Reading RG6 6DH, United Kingdom; School of Biological Sciences, University of Reading, Whiteknights, Reading RG6 6DH, United Kingdom; School of Biological Sciences, University of Reading, Whiteknights, Reading RG6 6DH, United Kingdom; School of Biological Sciences, University of Reading, Whiteknights, Reading RG6 6DH, United Kingdom; School of Biological Sciences, University of Reading, Whiteknights, Reading RG6 6DH, United Kingdom; School of Biological Sciences, University of Reading, Whiteknights, Reading RG6 6DH, United Kingdom

## Abstract

Understanding the structures of protein complexes is pivotal for breakthroughs in health, agriculture, bioengineering, and beyond. MultiFOLD2 and ModFOLDdock2 are leading servers for protein quaternary structure prediction and model quality assessment, respectively. MultiFOLD2 includes integrated stoichiometry prediction for quaternary structures and improved sampling and scoring, leading to high performance in continuous independent benchmarks such as CAMEO. ModFOLDdock2 uses a hybrid consensus approach to generate global and local quality scores for predicted quaternary structures. ModFOLDdock2 is integrated with MultiFOLD2 while also being available as a stand-alone server, enabling the independent evaluation of quaternary structure models from any source. Both servers have been independently rigorously evaluated, demonstrating high performance and ranking among the top servers in their respective categories in the recent CASP16 experiment. The MultiFOLD2 and ModFOLDdock2 servers are freely accessible through user-friendly web interfaces at https://www.reading.ac.uk/bioinf/.

## Graphical Abstract

**Graphical Abstract:**
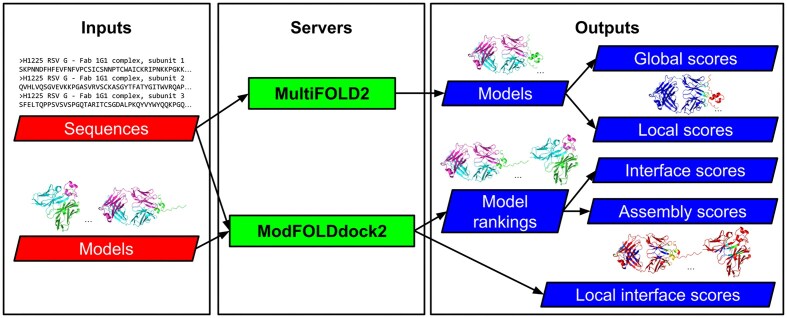

## Introduction

Following the success of AlphaFold2 in the CASP14 experiment [1], protein tertiary structures can be modelled with near-experimental accuracy. Therefore, since the CASP15 experiment, the prominent focus has been on developing methods for modelling protein quaternary structures and assessing their quality. For CASP15, we developed two complementary servers, MultiFOLD (https://www.reading.ac.uk/bioinf/MultiFOLD/) for tertiary and quaternary structure prediction, and ModFOLDdock (https://www.reading.ac.uk/bioinf/ModFOLDdock/) for estimating the accuracy of multimeric models [2–4]. In this paper, we outline the major updates to the MultiFOLD and ModFOLDdock servers, describe their functionality, and report on their performance in the most recent major independent international benchmarks, CASP16 and CAMEO.

MultiFOLD2 and ModFOLDdock2 were both independently benchmarked by the CASP16 assessors. MultiFOLD2 is also continuously evaluated by the CAMEO resource [5]. In CASP16, one of the major changes to the experiment was the phased release of multimer targets. In Phase 0, servers were required to predict the stoichiometry of targets, as this information was not initially provided. In addition, the CAMEO resource does not provide stoichiometry information when submitting targets to the participating servers. Therefore, for MultiFOLD2, one of our major developments was to provide the server with more robust stoichiometry prediction. Furthermore, the MultiFOLD2 server includes improved sampling, with the addition of models from AlphaFold2-Multimer, including dropout [6], RoseTTAFold2 [7], and RoseTTAFold-All-Atom [8], as well as improved model scoring using ModFOLDdock2.

MultiFOLD2 outperforms AlphaFold3 [9] (AF3) overall in the continuous CAMEO benchmark. In CASP16, MultiFOLD2 was ranked sixth among all server methods on medium and hard targets and was the top-ranked server method on the very hardest domain targets according to GDT_TS. MultiFOLD2 surpassed the AlphaFold2 (ColabFold) baseline server in the multimer category. Additionally, MultiFOLD2 outperformed AF3 on Phase 0 multimer targets based on official lDDT scores.

The ModFOLDdock2 server uses a hybrid consensus approach for producing both global and local (interface residue) quality scores for predicted quaternary structures. ModFOLDdock2 is integrated with MultiFOLD2, but we also make it available as a stand-alone server to allow for independent evaluation of user models from any source. In brief, the main differences from the original ModFOLDdock server were the addition of new scores and a neural network to predict local scores.

ModFOLDdock2 achieved first place in CASP16 for scoring the global and local interfaces of quaternary structure models, and its variants performed well across the board. As a result, we were invited to present our methods at the CASP16 conference in December 2024.

Following rigorous independent evaluation of MultiFOLD2 and ModFOLDdock2 in CASP16 and CAMEO, we have provided intuitive web interfaces for them and made them freely available to all, for the benefit of non-expert predictors in the worldwide life science community.

## Materials and methods

The core methodological aspects of the MultiFOLD2 and ModFOLDdock2 servers are summarized below. For further details and specific parameters, please refer to our previous papers, CASP16 abstracts and presentations (https://predictioncenter.org/casp16/). Supplementary Fig. S1 shows a flowchart of the data and process in MultiFOLD2 and its integration with ModFOLDdock2.

### MultiFOLD2

The three primary stages of the MultiFOLD2 protocol are sampling, scoring, and refinement. In the absence of user-provided stoichiometry information for multimer predictions, MultiFOLD2 will compute the stoichiometry prior to sampling. First, initial 3D models are generated using LocalColabFold [10] (https://github.com/YoshitakaMo/localcolabfold) with templates. If templates cannot be found from the target sequences, then Foldseek [11] is used with each chain of the top initial 3D model to find templates for each subunit and the stoichiometry for each template is determined. If templates are found for all subunits, then the most frequent stoichiometries from all templates are assigned to the target sequences and then used in subsequent modelling. If templates are still not found for a sequence, then QUEEN [12] is used to assign stoichiometry directly from the target sequences.

Following stoichiometry prediction, model sampling is then carried out using two different versions of LocalColabFold and RoseTTAFold2 (with and without dropout), and RoseTTAFold-All-Atom, generating up to 45 initial 3D models. In the second step of the process, the models are scored and ranked using ModFOLDdock2S (see below), which produces the global quality scores for ranking models. In the final step, the top 5 ModFOLDdock2S selected models are used as input templates for our AlphaFold2-Multimer_Refine [13] protocol, generating up to 55 models. For each model, the rankings and predicted per-residue quality scores (plDDT*100) from LocalColabFold are added to the B-factor column for each set of atom records. The MultiFOLD2 server is available at https://www.reading.ac.uk/bioinf/MultiFOLD/. MultiFOLD2 is also available as a docker image: https://hub.docker.com/r/mcguffin/multifold2.

### ModFOLDdock2

Using a hybrid consensus approach, the ModFOLDdock2 server produces global and local (interface residue) quality scores for predicted quaternary structures. Like the original version of the server, there are three variants to choose from: ModFOLDdock2, ModFOLDdock2R, and ModFOLDdock2S. Each of the ModFOLDdock2 variants uses specific combinations of global and local scores, which are calculated using the output from 12 individual scoring methods, including QS-bestJury, DockQ-waveJury, TM-scoreJury, Oligo-GDTJury, lDDTJury, CADJury, PatchQSJury, and PatchDockQJury, which use scores from OpenStructure [14] version 2.7, and VoroMQA [15], VoroIF [16], CDA [17–19], and ModFOLDIA [2].

The ModFOLDdock2 variant requires multiple input models and is optimized to generate quality estimates that correlate linearly with observed quality scores. The ModFOLDdock2R variant also needs multiple input models, but it is optimized to produce predicted scores for ranking, where the highest-ranked models (top 1) should have higher observed overall accuracy. The ModFOLDdock2S variant is designed to work with a single input model, using sets of reference multimer models generated using our MultiFOLD2 method (see above). Each input model is then compared individually against the reference set using the individual scoring methods described above. In addition, the ModFOLDdock2S interface residue scoring uses a Neural Network (NN) to predict the mean of the local lDDT, CAD, PatchQS, and PatchDockQ scores. The ModFOLDdock2 server is available at https://www.reading.ac.uk/bioinf/ModFOLDdock/. ModFOLDdock2 is also available via the MultiFOLD2 docker image: https://hub.docker.com/r/mcguffin/multifold2.

## Results and discussion

**Figure 1.**
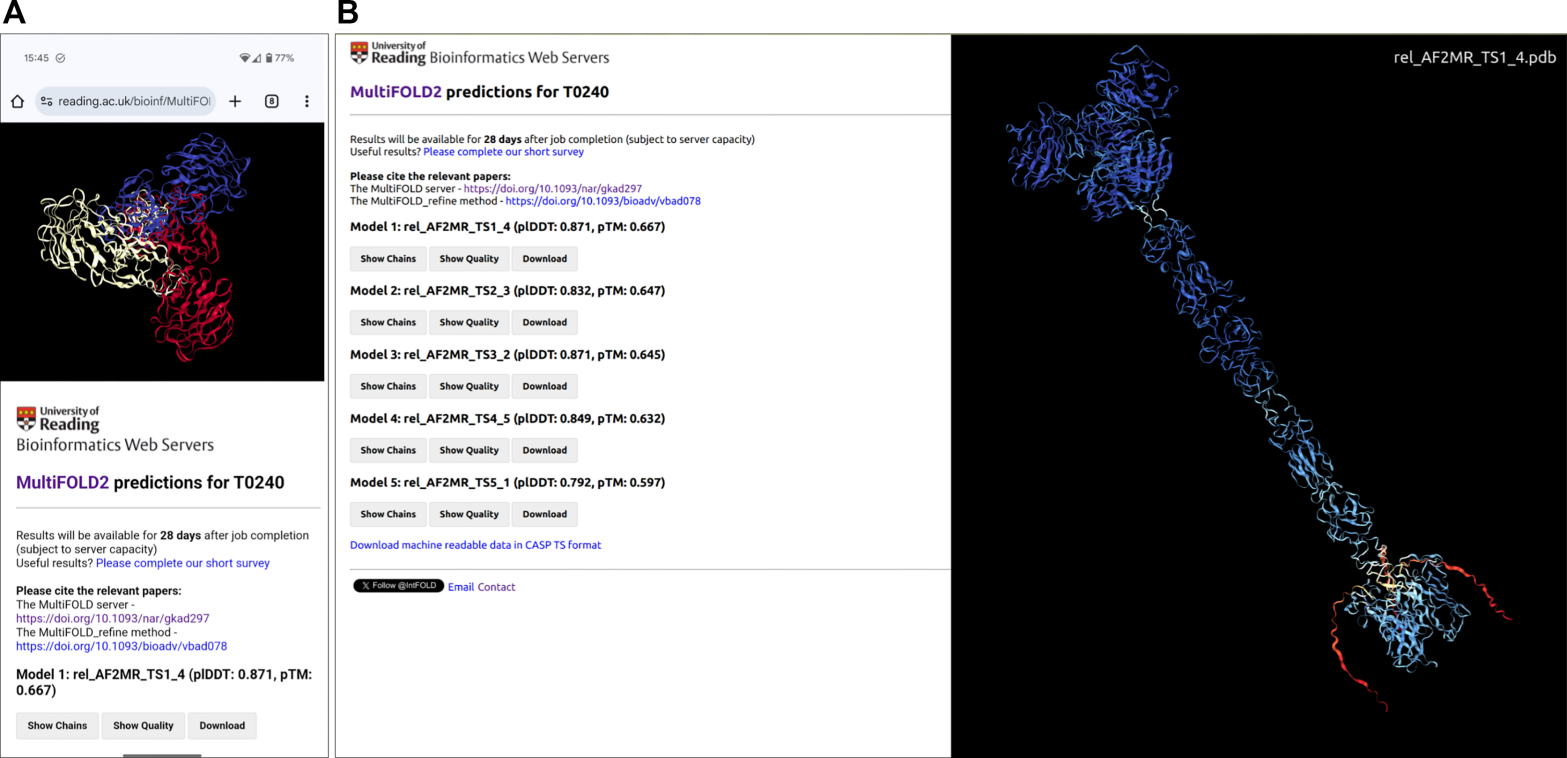
MultiFOLD2 server results pages for CASP16 target T0240 with correctly predicted stoichiometry A3, viewed using mobile and desktop browsers. (**A**) Screenshot from an Android phone browser in portrait orientation. The top model is coloured by chain identifier, which can be selected using the ‘Show Chains’ button. (**B**) Screenshot from an Ubuntu laptop browser. The top model is rotated and coloured by predicted local model quality (the per-residue plDDT scores) using the ‘Show Quality’ button.

Both servers integrate aspects of each other and present their output consistently to users (Fig. 1 and Supplementary Fig. S2). The resulting models of protein complexes can be viewed interactively in 3D directly within the web browser. Machine-readable data files are also provided by each server, which comply with the CASP data standards for TS and QA formats.

### MultiFOLD2 server inputs and outputs

MultiFOLD2 users are required to input the sequence or multiple sequences for their target complex in FASTA format. Optionally, they can include their name and email address to receive a notification of their job completion, or they may simply bookmark the results page link provided after submission. Providing stoichiometry information for the complex, if known, is recommended. In cases where stoichiometry is unknown, MultiFOLD2 will predict this using known template structures if available or directly from the sequences.

The MultiFOLD2 results pages show the top 5 predicted 3D models, ranked by decreasing predicted global model quality scores, which can be viewed interactively in any standard desktop or mobile web browser (Fig. 1). The results page also includes buttons for each model, allowing users to colour models by chain identifier or predicted local quality, and to download models in PDB format. Machine-readable model files are also provided in CASP TS format.

### ModFOLDdock2 server inputs and outputs

ModFOLDdock2 users must input the target protein sequences in FASTA format, the stoichiometry of the target complex, and a single 3D model for evaluation. Alternatively, users may upload a tarball containing multiple alternative models, and optionally, they may provide a name for their protein sequence and an email address. The server form offers three variants with different component scoring methods: ModFOLDdock2, with predicted global scores optimized for positive linear correlations with observed scores; ModFOLDdock2R, with global scores optimized for ranking the best models; and ModFOLDdock2S, for scoring single models.

The ModFOLDdock2 results pages rank models based on predicted global assembly and interface quality scores (Supplementary Fig. S2). Ranked protein complex models can be viewed interactively in 3D within a standard desktop or mobile web browser, similar to MultiFOLD2. The table also provides buttons for colouring models by chain identifier or predicted local interface quality. Users can download models as PDB files with predicted local interface scores in the B-factor column. Machine-readable model files that comply with the latest CASP QA (QMODE2) format are also available.

### Independent benchmarking

Both MultiFOLD2 and ModFOLDdock2 were independently assessed in the recent blind CASP16 experiment. Both servers ranked among the top servers in their respective categories. Additionally, MultiFOLD2 is continuously independently benchmarked by the CAMEO resource, although there is currently no similar CAMEO facility for evaluating quality assessment methods like ModFOLDdock2. A summary of the results from CAMEO and CASP16 follows.

**Figure 2.**
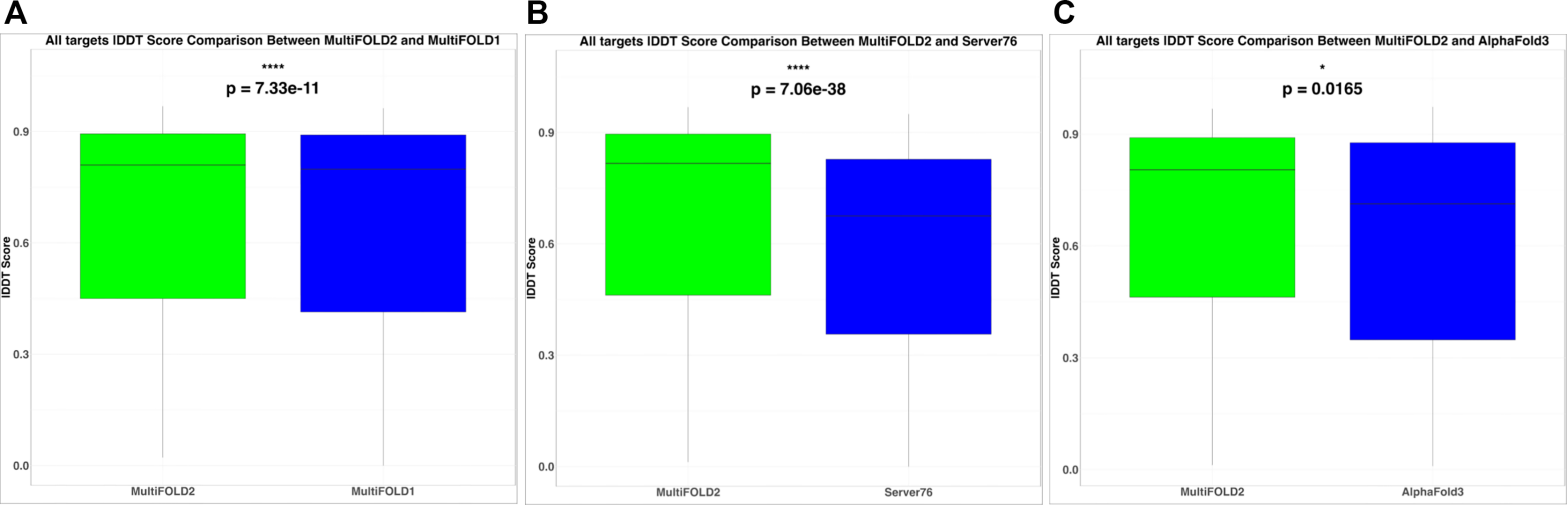
Performance comparison of MultiFOLD2 versus the other servers participating in the independent blind CAMEO BETA benchmark according to the lDDT score. Common subset comparisons for all target types (monomers, homomers, and heteromers) were made between servers using *n* targets. (**A**) MultiFOLD2 versus MultiFOLD1 with *n*= 536 targets. (**B**) MultiFOLD2 versus Server76 with *n*= 544 targets. (**C**) MultiFOLD2 versus AF3 with *n*= 596 targets. The *P*-values are for the Wilcoxon signed-rank test. Data collection was from 11 May 2024 (except for AF3, which was from 18 May 2024) until 18 January 2025. Data are from https://beta.cameo3d.org/complete-modeling/.

*CAMEO results summary*. Figure 2 shows that MultiFOLD2 significantly outperforms all servers currently participating in CAMEO BETA according to the lDDT official score data. This includes our previous version of MultiFOLD, the anonymous Server 76, and AF3 based on common target subset analyses of the lDDT scores for models for all target types.

Supplementary Figs S3 and S4 show the relative performance according to the multimer targets (homomers and heteromers). MultiFOLD2 significantly outperforms all other servers on all multimer targets according to the lDDT and the QS official score data (Supplementary Fig. S3). The data for heteromers show that MultiFOLD2 performance is significantly better than the original version of MultiFOLD and Server 76, and similar to AF3 according to the lDDT scores. For homomer targets, MultiFOLD2 significantly outperforms all three methods—MultiFOLD, Server 76, and AF3 (Supplementary Fig. S4).

*CASP16 results summary*. MultiFOLD2 was the top-ranked server group in CASP16 according to the assessment of the hardest domain targets by GDT_TS (Supplementary Table S1). Furthermore, MultiFOLD2 ranked as the sixth best server on both the medium and hard domains, outperforming the AF2 (ColabFold) and AF3 baselines by GDT_TS (Supplementary Table S2).

In CASP16, many server groups used manually predicted AF3 models for their multimer predictions in all target phases, due to the lack of AF3 code and the ability to automate it at the time. However, MultiFOLD2 ran as a pure server method throughout the CASP16 prediction season for Phase 0 (where no stoichiometry information was provided), with no integration of AF3 models. Supplementary Fig. S5 shows that MultiFOLD2 outperforms the AF3-server group method according to lDDT scores for the CASP16 Phase 0 multimer targets and performs similarly according to the QS score. The effect of target size on performance depends on the metric used. MultiFOLD2 performance is best on the smaller targets according to the QS-best and DockQ scores. However, the performance is best on medium-sized targets according to the lDDT score and TM score and on large targets according to the ICS score (Supplementary Fig. S6).

**Figure 3.**
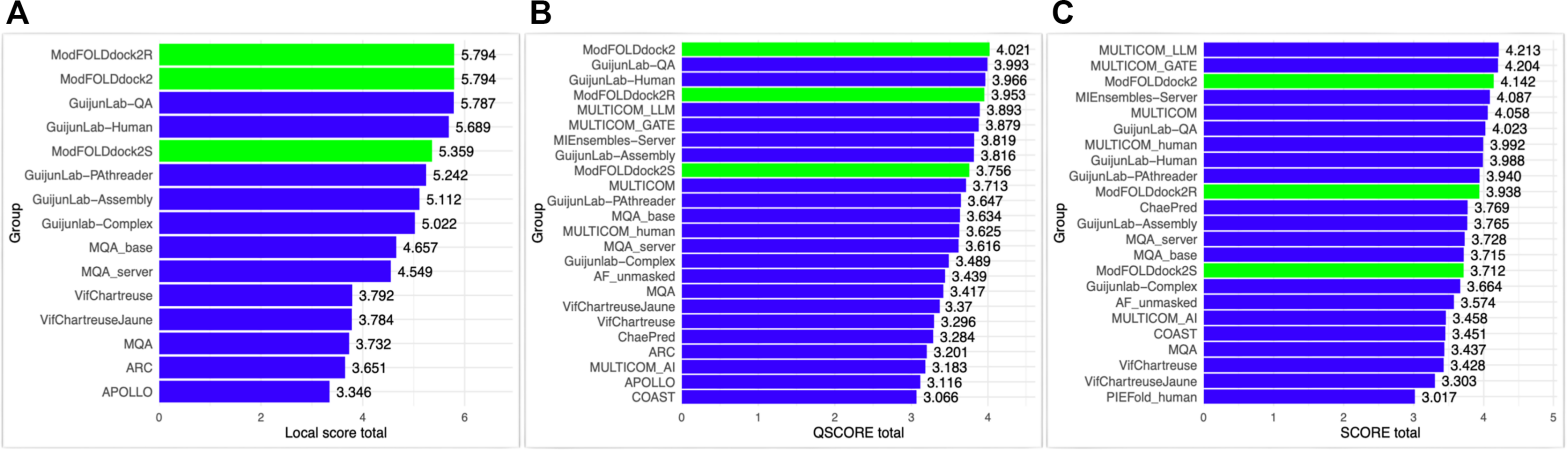
The performance of the ModFOLDdock2 server methods in CASP16 versus other participating predictor groups according to the official scores. (**A**) Performance of servers in the prediction of local interface residue accuracy. The local score total is calculated using an assessor-like formula but for total average local scores [0.5*Pearson(PatchDockQ) + 0.5*Spearman(PatchDockQ) + AUC(PatchDockQ) + 0.5*Pearson(PatchQS) + 0.5*Spearman(PatchQS) + AUC(PatchQS) + 0.5*Pearson(CAD) + 0.5*Spearman(CAD) + AUC(CAD) + 0.5*Pearson(lDDT) + 0.5*Spearman(lDDT) + AUC(lDDT)]. Ranks are tied for ModFOLDdock2 and ModFOLDdock2R because they use the same code for local scores. (**B**) Global interface (QSCORE) prediction performance. The QSCORE score total is calculated using [0.5*Pearson(DockQ-wave) + 0.5*Spearman(DockQ-wave) + AUC(DockQ-wave) + 1-Loss(DockQ-wave) + 0.5*Pearson(QS) + 0.5*Spearman(QS) + AUC(QS) + 1-Loss(QS)]. (**C**) Global fold (SCORE) prediction performance. The SCORE score total is calculated using [0.5*Pearson(GDT_TS) + 0.5*Spearman(GDT_TS) + AUC(GDT_TS) + 1-Loss(GDT_TS) + 0.5*Pearson(TM) + 0.5*Spearman(TM) + AUC(TM) + 1-Loss(QTM)]. Data are from https://predictioncenter.org/casp16/results.cgi?tr_type=accuracy.

ModFOLDdock2 was the best method in CASP16 for predicting both the global interface score (QSCORE) and the local (per-residue) interface accuracy of modelled protein complexes (Fig. 3). Furthermore, at least one of the ModFOLDdock2 server variants ranked within the top 5 groups across all metrics, including the global fold (SCORE) (Supplementary Table S3). ModFOLDdock2 local (QMODE2) score performance decreases as the target size increases and there are more interface residues to score (Supplementary Fig. S7). However, in terms of the global (QMODE1) scores, while the performance is better on small targets, there is less of an association between target size and performance according to the official metrics (Supplementary Fig. S8).

**Figure 4.**
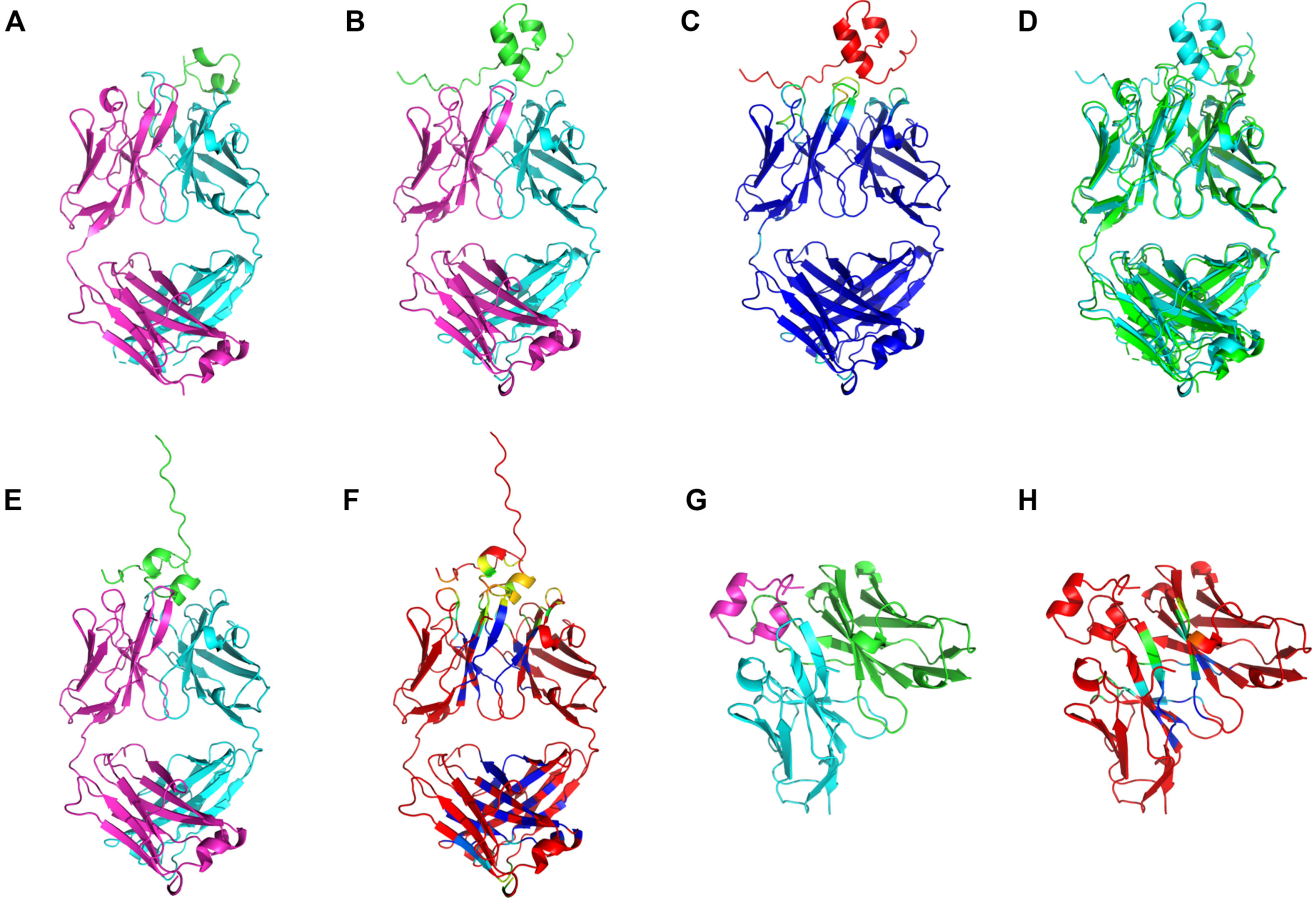
Examples of MultiFOLD2 and ModFOLDdock2 predictions for a heteromultimeric CASP16 target H0225 (Phase 0)/H1225 (Phase 1) with the stoichiometry A1B1C1. (**A**) The native structure of H0225/H1225 is coloured by chain identifiers. (**B**) The MultiFOLD2 top predicted model for H0225 (Phase 0) is also coloured by chain and shows the correct stoichiometry. (**C**) The MultiFOLD2 model is coloured by predicted local accuracy (plDDT) ranging in a spectrum from blue (high accuracy) to red (low accuracy). (**D**) Superposition of the MultiFOLD2 model (cyan) with the native structure for H0225/H1225 (green). (**E**) Top-ranked model by ModFOLDdock2 for H1225 coloured by chain identifier. Loss (the difference in quality between the best and selected models) according to each global score: GDT-TS = 0.051, TM score = 0.022, DockQ-Wave = 0.058, and QS score = 0.131. AUC (area under receiver operating characteristic curves) scores measuring the accuracy of local interface scoring: PatchDockQ = 0.829, PatchQS = 0.803, CAD = 0.855, and lDDT = 0.818. (**F**) Top-ranked model by ModFOLDdock2 for H1225 coloured by predicted interface residue accuracy from blue (high confidence of interface residue) to red (very low confidence, or non-interface residue). (**G**) Bottom-ranked model by ModFOLDdock2 for H1225 coloured by chain identifier. (**H**) Bottom-ranked model by ModFOLDdock2 for H1225 coloured by predicted interface residue accuracy ranging in a spectrum from blue (high confidence of interface residue) to red (very low confidence, or non-interface residue). Data are from https://predictioncenter.org/download_area/CASP16/.

Figure 4 shows examples of MultiFOLD2 predictions (Fig. 4B–D) and ModFOLDdock2 quality assessment of models (Fig. 4E–H) for the CASP16 target H0225 (Phase 0)/H1225 (Phase 1). MultiFOLD2 correctly predicted the stoichiometry and overall assembly of the H0225 complex (lDDT = 0.852, TM score = 0.927) apart from the position of the interacting peptide (shown in green, Fig. 4B). Figure 4C shows the MultiFOLD2 model coloured by local plDDT scores, indicating that MultiFOLD2 correctly identified the inaccurate position of the peptide, but was correctly confident about the overall assembly of the main chains. Figure 4E and F shows the top-ranked model (1/377) by ModFOLDdock2 for H1225, which has the correct overall assembly. ModFOLDdock2 confidently and accurately predicted the interacting main chain residues for the top model (Fig. 4F), while the server was appropriately less certain about the interacting residues for the peptide. Figure 4G and H shows the bottom-ranked model (377/377) by ModFOLDdock2 for H1122, which has the incorrect overall assembly and few correctly interacting residues, indicating that the server was appropriately less certain about this model.

## Conclusions

MultiFOLD2 and ModFOLDdock2 are state-of-the-art servers for protein complex prediction and quality assessment. MultiFOLD2 integrates stoichiometry prediction and improved sampling and scoring, leading to high performance. ModFOLDdock2 is integrated with MultiFOLD2 and can be used as a stand-alone server providing global and local quality scores for predicted quaternary structures using a hybrid consensus approach. Both servers have been rigorously evaluated and independently benchmarked, demonstrating their high performance and ranking among the top servers in their respective categories of CASP16 and CAMEO. These user-friendly servers are proven leading tools for the prediction and quality assessment of protein quaternary structure models and are freely available to the global life science community.

## Supplementary Material

gkaf336_Supplemental_File

## Data Availability

The data underlying this article are available in the article and in its online supplementary material. The MultiFOLD2 and ModFOLDdock2 servers are available at https://www.reading.ac.uk/bioinf/. The methods are also available to download as a docker image: https://hub.docker.com/r/mcguffin/multifold2.

## Notes

present address: department of structural molecular biology (smb), ucl darwin building, gower street, london wc1e 6bt, united kingdom
