# Supplementary material for: Prediction and quality assessment of protein quaternary structure models using the MultiFOLD2 and ModFOLDdock2 servers
Source: Nucleic Acids Res. 2025 Apr 25;53(W1):W472–7. doi: 10.1093/nar/gkaf336 (PMC12230669; doi:10.1093/nar/gkaf336)

Supplementary Figure S1. Flowchart of MultiFOLD2 integrating sampling using LocalColabFold and RoseTTAFold variants and scoring using ModFOLDdock2. MultiFOLD2 is also used in the ModFOLDdock2S variant to generate reference sets of models.

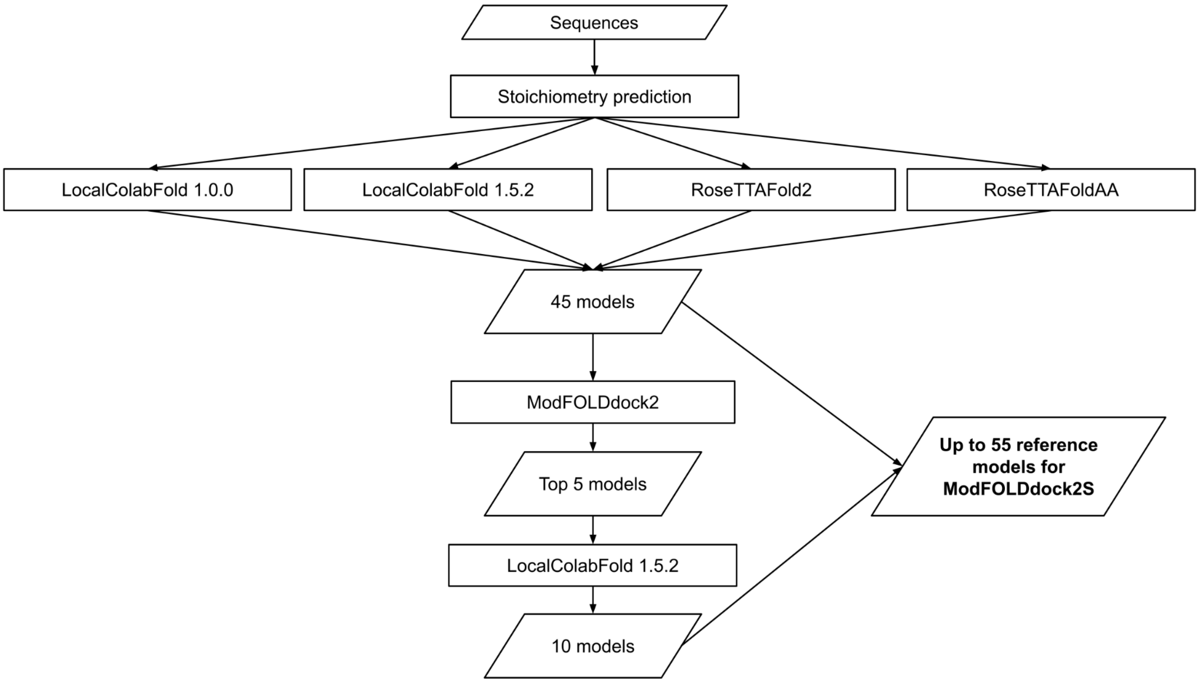

Supplementary Figure S2. ModFOLDdock2R server results page for CASP16 target H1233 with the stoichiometry A2B2B2, viewed using mobile and desktop browsers (only the top few models out of 343 are shown in the example screenshots, but users may scroll down the page to view all of their models). **(A)** Screenshot from an Android phone browser in portrait orientation. The top model is coloured by predicted local interface model quality (the confidence score relates to the probability of the residue in the model being in the actual interface of the native structure) using the “Interface Quality” button. **(B)** Screenshot from an Ubuntu laptop browser. The top-ranked model is coloured by chain identifier which can be selected using the “Show Chains” button.

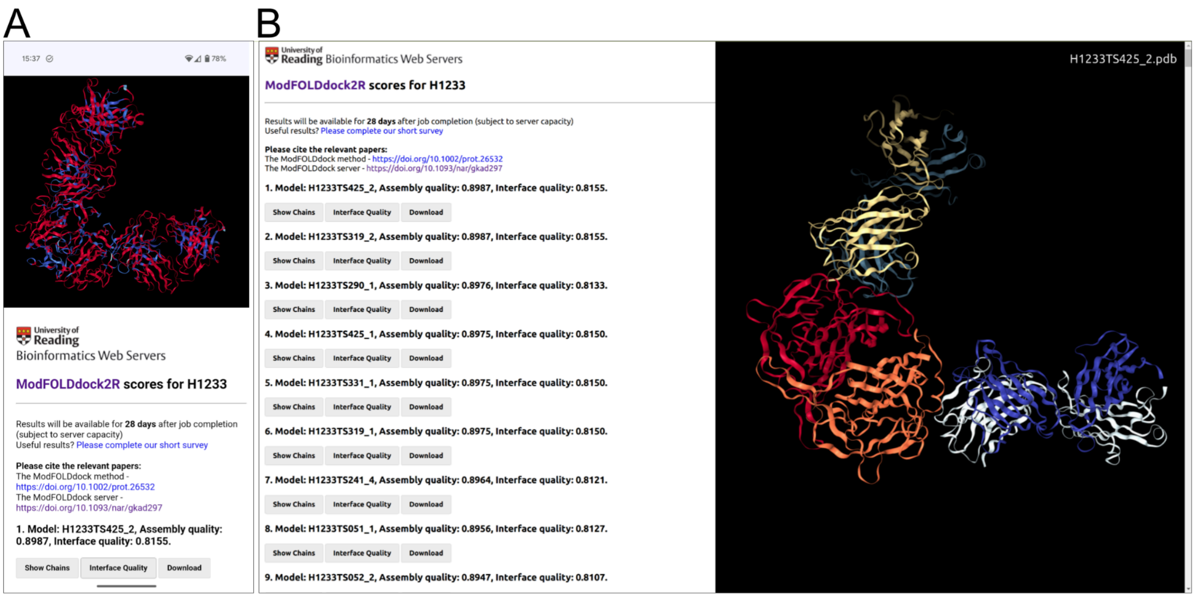

Supplementary Figure S3. Performance comparison of MultiFOLD2 versus the other servers participating in the independent blind CAMEO BETA benchmark. Common subset comparisons between servers were made for all multimer target types (homomers, heteromers) using *n* targets. **(A)** IDDT score performance, MultiFOLD2 vs MultiFOLD1 with *n*=390 targets. **(B)** IDDT score performance, MultiFOLD2 vs Server76 with n=377 targets. **(C)** IDDT score performance, MultiFOLD2 vs AlphaFold3 with *n*=437 targets. **(D)** QS score performance, MultiFOLD2 vs MultiFOLD1 with *n*=390 targets. **(E)** QS score performance, MultiFOLD2 vs Server76 with n=377 targets. **(F)** QS score performance, MultiFOLD2 vs AlphaFold3 with *n*=437 targets. The p-values are for the Wilcoxon signed-rank test. Data collection was from 11.05.2024 (except for AF3, which was from 18.05.2024) until 18.01.2025. Data are from: https://beta.cameo3d.org/complete-modeling/

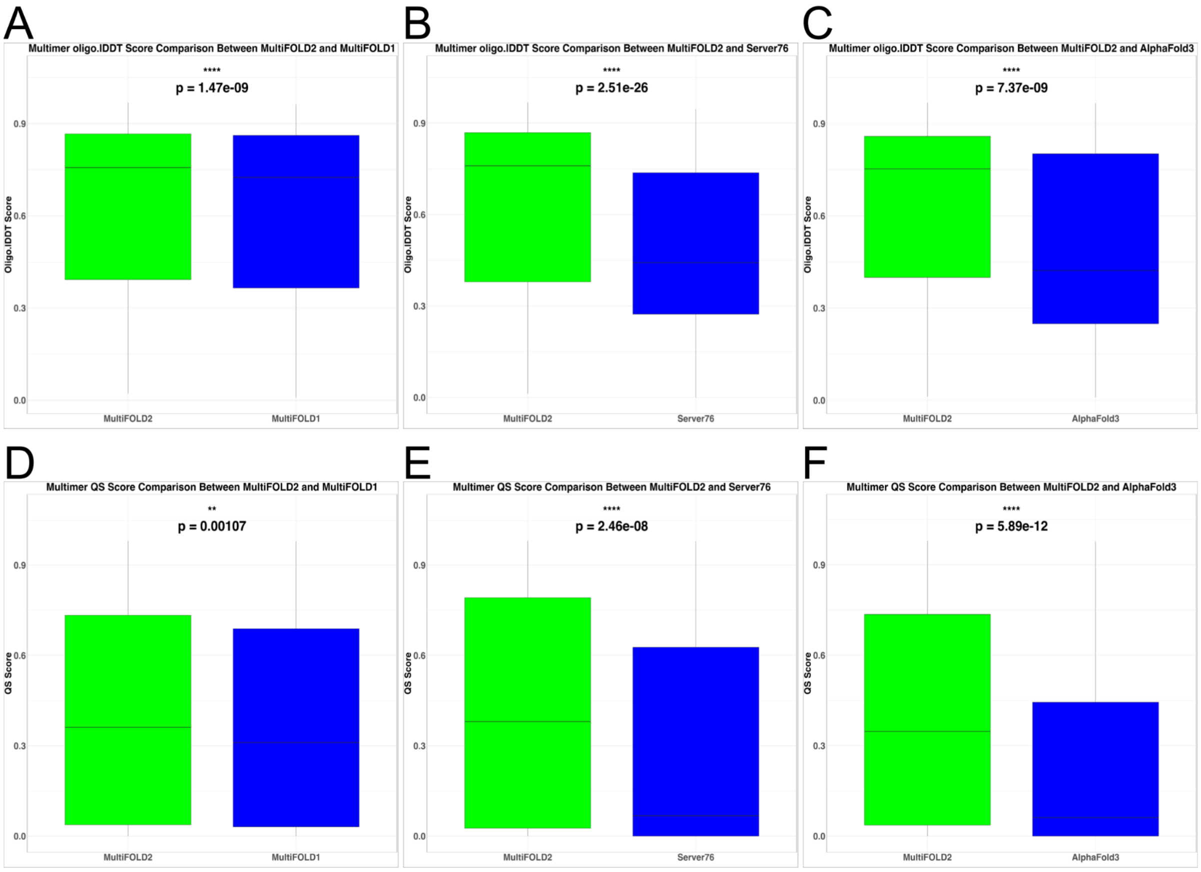

Supplementary Figure S4. Performance comparison of MultiFOLD2 versus the other servers participating in the independent blind CAMEO BETA benchmark. Common subset comparisons between servers were made for homomer targets (A-C) and heteromer targets (D-F) separately using *n* targets. **(A)** IDDT score performance for homomers, MultiFOLD2 vs MultiFOLD1 with *n*=152 targets. **(B)** IDDT score performance for homomers, MultiFOLD2 vs Server76 with n=188 targets. **(C)** IDDT score performance for homomers, MultiFOLD2 vs AlphaFold3 with *n*=164 targets. **(D)** IDDT score performance for heteromers, MultiFOLD2 vs MultiFOLD1 with *n*=238 targets. **(E)** IDDT score performance for heteromers, MultiFOLD2 vs Server76 with n=189 targets. **(F)** IDDT score performance for heteromers, MultiFOLD2 vs AlphaFold3 with *n*=273 targets. The p-values are for the Wilcoxon signed-rank test. Data collection was from 11.05.2024 (except for AF3, which was from 18.05.2024) until 18.01.2025. The QS scores were 0 for the AF3 homomers, so it is not as useful a comparison metric for these subsets. However, the MultiFOLD2 performance for homomers is also significantly higher than all other methods according to the QS score ($p<0.05$). For heteromomers, MultiFOLD2 performance is significantly higher than MultiFOLD ($p<0.01$) and similar for the other methods. Data are from: https://beta.cameo3d.org/complete-modeling/

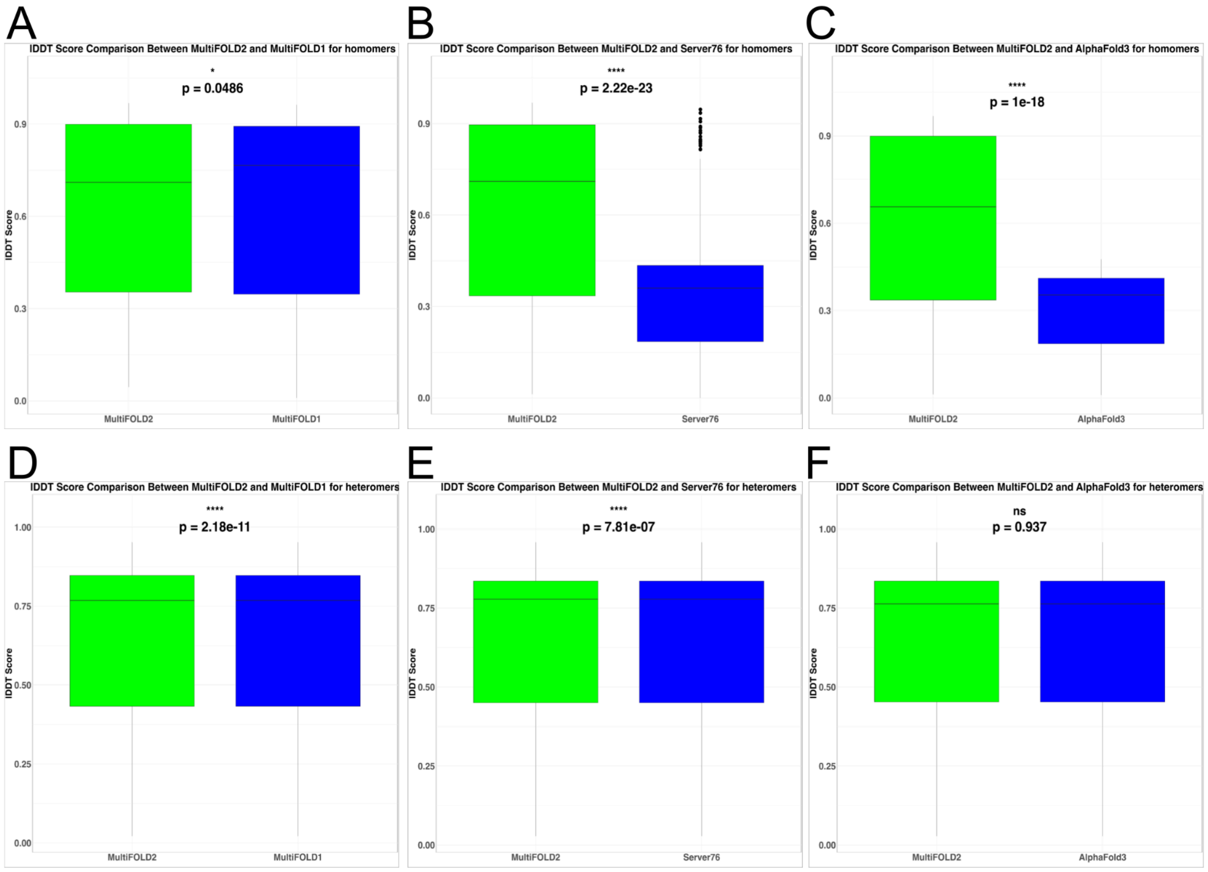

Supplementary Table S1. MultiFOLD2 performance on the CASP16 hardest (FM) domains compared to all other participating server methods. Z-score based relative group performance. Ranking on the models designated as "1". Server groups on 'all groups' + 'server only' targets. The ranking of groups is based on the sum of Z-scores (>0.0) for hard domains according to GDT-TS. Data are from: https://predictioncenter.org/casp16/zscores_final.cgi?model_type=first&gr_type=server_only&formula=gdt_ts&fm=on).

GR name	SUM Zscore (>-2.0)	Rank SUM Zscore (>-2.0)	SUM Zscore (>0.0)	Rank SUM Zscore (>0.0)
MultiFOLD2	6.1737	1	6.7069	1
Yang-Multimer	4.0649	2	6.3674	2
colabfold_baseline	2.9593	3	5.9823	3
CSSB_server	0.5265	9	4.9253	4
Yang-Server	1.9972	5	4.7697	5
MRAFold	0.5708	8	4.3121	6
DeepFold-server	2.1541	4	4.3005	7
falcon2	0.4563	10	4.1701	8
GuijunLab-Assembly	-0.8085	15	3.7765	9
Guijunlab-Complex	0.4225	11	3.655	10
colabfold	0.204	12	3.2377	11
MULTICOM_GATE	1.718	6	3.2098	12
MULTICOM_LLM	-0.7338	14	3.1688	13
milliseconds	-1.8992	20	3.036	14
MULTICOM_AI	-1.1453	17	2.8718	15
kiharalab_server	0.8152	7	2.5326	16
NKRNA-s	-5.7681	26	2.2523	17
OpenComplex_Server	-4.2028	23	2.241	18
Zheng-Multimer	-1.5305	19	2.2354	19
Zheng-Server	-0.9128	16	2.2328	20
MIEnsembles-Server	-1.3649	18	2.2001	21
GuijunLab-PAthreader	-0.3934	13	2.0076	22
MQA_server	-4.9942	24	1.5826	23
Unicorn	-5.5172	25	1.4026	24
ptq	-2.5547	22	1.3931	25
AF3-server	-2.3763	21	1.2572	26
ShanghaiTech-server	-7.3786	28	1.1157	27

GHZ-ISM	-5.8768	27	1.043	28
XGroup-server	-11.5177	29	0.4823	29
profold2	-13.9465	30	0.0535	30
Cerebra_server	-14.5569	31	0	31

Supplementary Table S2. MultiFOLD2 performance on the CASP16 medium and hard (TBM/FM+FM) domains compared to all other participating server methods. Z-score based relative group performance. Ranking on the models designated as "1". Server groups on 'all groups' + 'server only' targets. The ranking of groups is based on the sum of Z-scores (>0.0) for hard domains according to GDT-TS. Data are from: https://predictioncenter.org/casp16/zscores_final.cgi?model_type=first&gr_type=server_only&formula=gdt_ts&&tbmfm=on&fm=on).

GR name	SUM Zscore (>-2.0)	Rank SUM Zscore (>-2.0)	SUM Zscore (>0.0)	Rank SUM Zscore (>0.0)
Yang-Server	18.9275	1	31.5709	1
falcon2	3.7102	7	28.4101	2
Yang-Multimer	12.0301	2	25.8063	3
Unicorn	5.7198	4	23.2078	4
GHZ-ISM	5.3603	5	22.8482	5
MultiFOLD2	7.7201	3	22.6797	6
MQA_server	-8.2977	19	21.4401	7
NKRNA-s	-5.8049	18	21.2878	8
MRAFold	2.9262	9	19.9492	9
MULTICOM_LLM	4.2402	6	19.4326	10
DeepFold-server	-15.17	22	19.2012	11
kiharalab_server	-1.337	13	19.1354	12
MULTICOM_AI	0.8625	12	19.033	13
ptq	-4.5736	16	18.7149	14
colabfold_baseline	-26.0269	25	18.6053	15
CSSB_server	-18.1419	23	17.7439	16
Zheng-Server	1.9245	10	17.4962	17
MULTICOM_GATE	3.6112	8	17.4479	18
Zheng-Multimer	-1.3543	14	16.8224	19
MIEnsembles-Server	1.0019	11	16.621	20
GuijunLab-Assembly	-10.1304	21	14.5654	21
milliseconds	-18.4282	24	14.3913	22
Guijunlab-Complex	-5.193	17	14.2181	23
AF3-server	-1.9252	15	13.6205	24

colabfold	-35.3199	27	12.986	25
GuijunLab-PAthreader	-8.4629	20	12.731	26
ShanghaiTech-server	-31.8247	26	11.1255	27
OpenComplex_Server	-62.484	28	6.7443	28
XGroup-server	-70.4808	29	6.2152	29
Cerebra_server	-94.9168	30	1.8736	30
colabfold_foldseek	-105.3212	32	0.6788	31
profold2	-104.9955	31	0.0535	32
APOLLO	-106.0693	33	0	33
ARC	-106.0693	33	0	33
COAST	-106.8946	35	0	33

Supplementary Figure S5. MultiFOLD2 performance on the CASP16 Phase 0 targets compared to all other participating server methods. In Phase 0, servers are not provided with the target stoichiometry at the time of submission. It should be noted that some of the groups listed as servers in CASP16 were not "true" servers as they had significant manual input, including for the stoichiometry prediction (e.g. DeepMind did not participate officially and the AF3-server lacked stoichiometry prediction, therefore the Elofsson group used the AF3 server to make manual predictions and submitted the highest scoring complexes). **(A)** Cumulative Phase 0 lDDT scores for each server group. **(B)** Cumulative Phase 0 QS scores for each server group. Data are from: https://predictioncenter.org/casp16/results.cgi?tr_type=multimer&phase=0&groups_id=&model=1

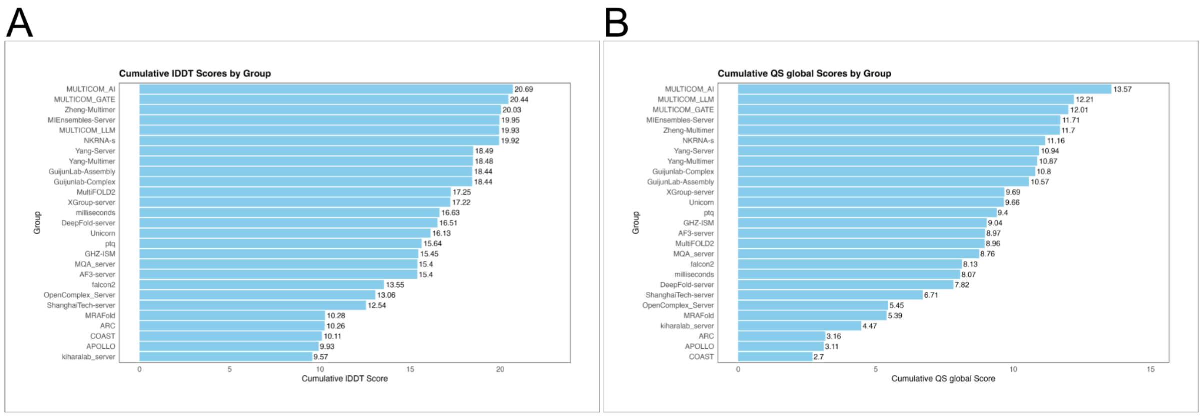

Supplementary Figure S6. MultiFOLD2 performance on the 25 CASP16 Phase 0 multimer targets according to target size (12 small targets <1000 amino acids, 6 medium targets 1000-2000 amino acids, 7 large targets >2000 amino acids).

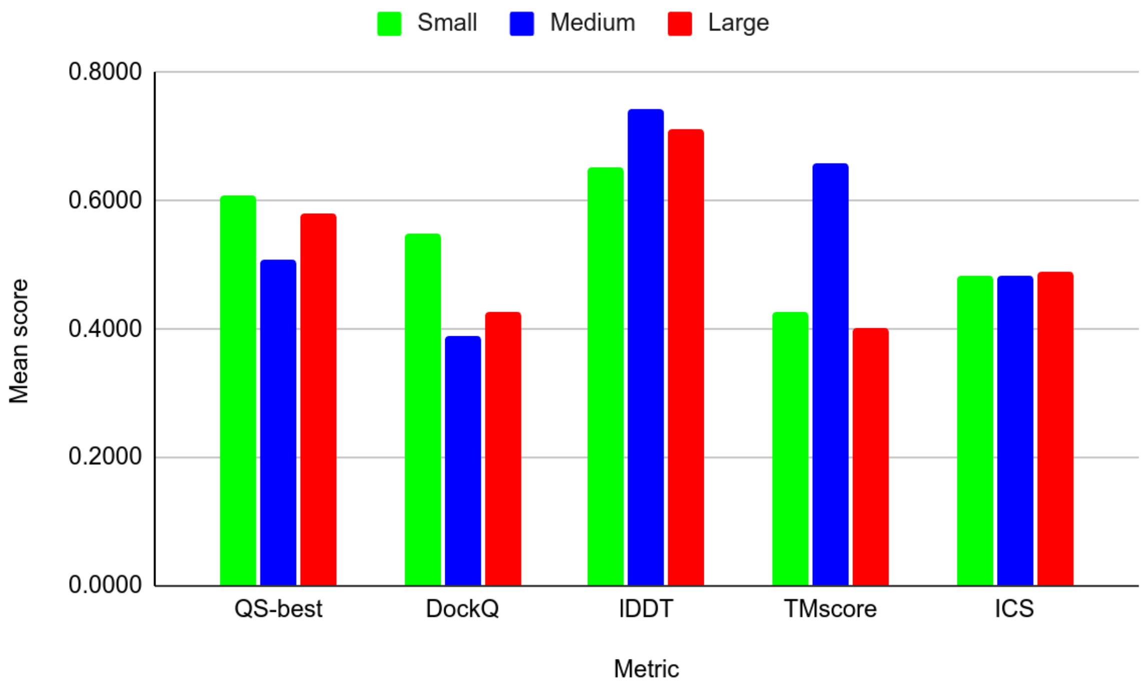

Supplementary Table S3. Summary of ModFOLDdock performance in CASP16 in terms of **(A)** Local score (QMODE2) and **(B)** Global (QMODE1) relative rankings versus all participating groups. Ranks are tied for ModFOLDdock2 and ModFOLDdock2R local scores because they use the same code. Cells are coloured green where the ModFOLDdock2 variant ranked within the top 5 groups overall according to that metric. Data are from: https://predictioncenter.org/casp16/results.cgi?tr_type=accuracy

<table>
<tr><th colspan="5">A) Local score rankings (QMODE2)</th></tr>
<tr><td></td><td></td><td colspan="3">PatchQS</td></tr>
<tr><td>Method</td><td>group_id</td><td>rank by roc_auc</td><td>rank by spearman_r</td><td>rank by pearson_r</td></tr>
<tr><td>ModFOLDdock2</td><td>441</td><td rowspan="2">**1st**</td><td rowspan="2">**1st**</td><td rowspan="2">**1st**</td></tr>
<tr><td>ModFOLDdock2R</td><td>27</td></tr>
<tr><td>ModFOLDdock2S</td><td>74</td><td>**4th**</td><td>**4th**</td><td>**4th**</td></tr>
<tr><td></td><td></td><td colspan="3">PatchDockQ</td></tr>
<tr><td>Method</td><td>group_id</td><td>rank by roc_auc</td><td>rank by spearman_r</td><td>rank by pearson_r</td></tr>
<tr><td>ModFOLDdock2</td><td>441</td><td rowspan="2">**1st**</td><td rowspan="2">**1st**</td><td rowspan="2">**1st**</td></tr>
<tr><td>ModFOLDdock2R</td><td>27</td></tr>
<tr><td>ModFOLDdock2S</td><td>74</td><td>**4th**</td><td>**4th**</td><td>**4th**</td></tr>
<tr><td></td><td></td><td colspan="3">CAD</td></tr>
<tr><td></td><td>group_id</td><td>rank by roc_auc</td><td>rank by spearman_r</td><td>rank by pearson_r</td></tr>
<tr><td>ModFOLDdock2</td><td>441</td><td rowspan="2">**2nd**</td><td rowspan="2">**2nd**</td><td rowspan="2">**3rd**</td></tr>
<tr><td>ModFOLDdock2R</td><td>27</td></tr>
</table>

ModFOLDdock2S	74	7th	**5th**	**5th**
		lDDT		
Method	group_id	rank by roc_auc	rank by spearman_r	rank by pearson_r
ModFOLDdock2	441	**3rd**	**3rd**	**3rd**
ModFOLDdock2R	27			
ModFOLDdock2S	74	7th	6th	**5th**

B) Global score rankings (QMODE1)				
		QS-best (Interface)		
Method	group_id	rank by roc_auc	rank by loss	rank by pearson_r
ModFOLDdock2	441	**1st**	**3rd**	**3rd**
ModFOLDdock2R	27	**2nd**	**2nd**	6th
ModFOLDdock2S	74	**5th**	**4th**	**4th**
		DockQ-Wave (Interface)		
Method	group_id	rank by roc_auc	rank by loss	rank by pearson_r
ModFOLDdock2	441	**1st**	**2nd**	**2nd**
ModFOLDdock2R	27	**3rd**	**1st**	**5th**
ModFOLDdock2S	74	20th	14th	6th
		TM-score (Fold)		
Method	group_id	rank by roc_auc	rank by loss	rank by pearson_r
ModFOLDdock2	441	**1st**	7th	**3rd**
ModFOLDdock2R	27	8th	8th	8th
ModFOLDdock2S	74	19th	19th	12th
		Oligo-GDT-TS (Fold)		
Method	group_id	rank by roc_auc	rank by loss	rank by pearson_r
ModFOLDdock2	441	**4th**	11th	**3rd**
ModFOLDdock2R	27	11th	8th	7th
ModFOLDdock2S	74	18th	16th	12th

Supplementary Figure S7. ModFOLDdock2 local score (QMODE2) performance on the CASP16 EMA targets according to target size (20 small targets <1000 amino acids, 9 medium targets 1000-2000 amino acids, 10 large targets >2000 amino acids). The performance according to each metric was measured by (Pearson + Spearman + AUC)/3.

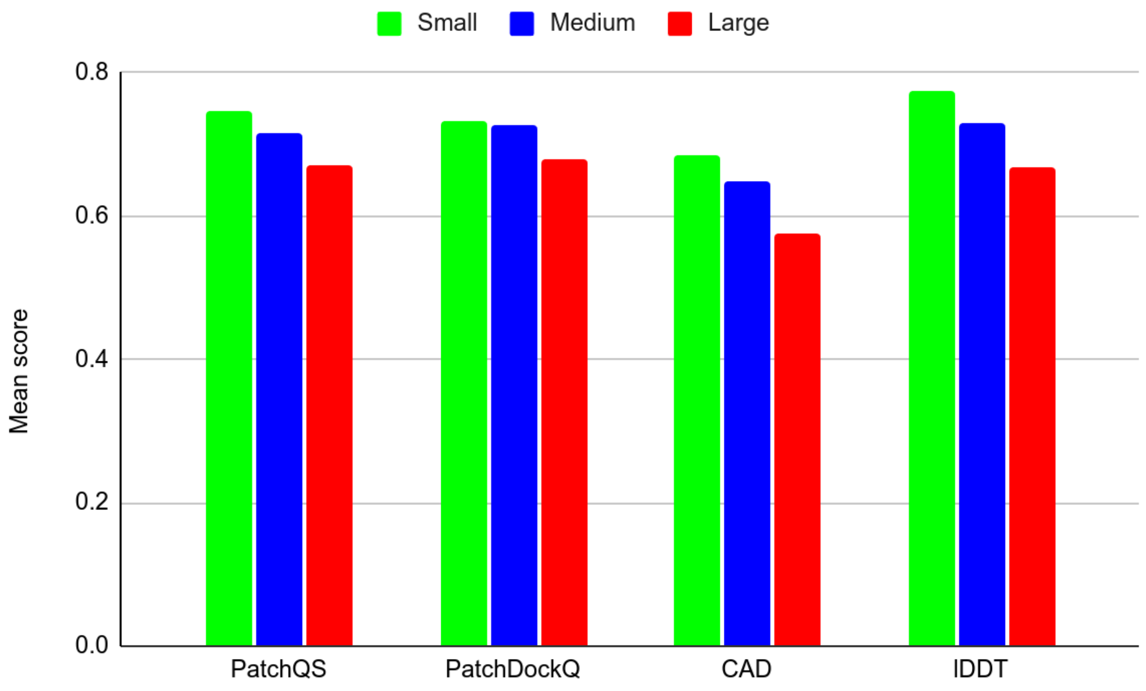

Supplementary Figure S8. ModFOLDdock2 global score (QMODE1) performance on the CASP16 EMA targets according to target size (20 small targets <1000 amino acids, 9 medium targets 1000-2000 amino acids, 10 large targets >2000 amino acids). The performance according to each metric was measured by (Pearson + Spearman + AUC +(1-Loss))/4.

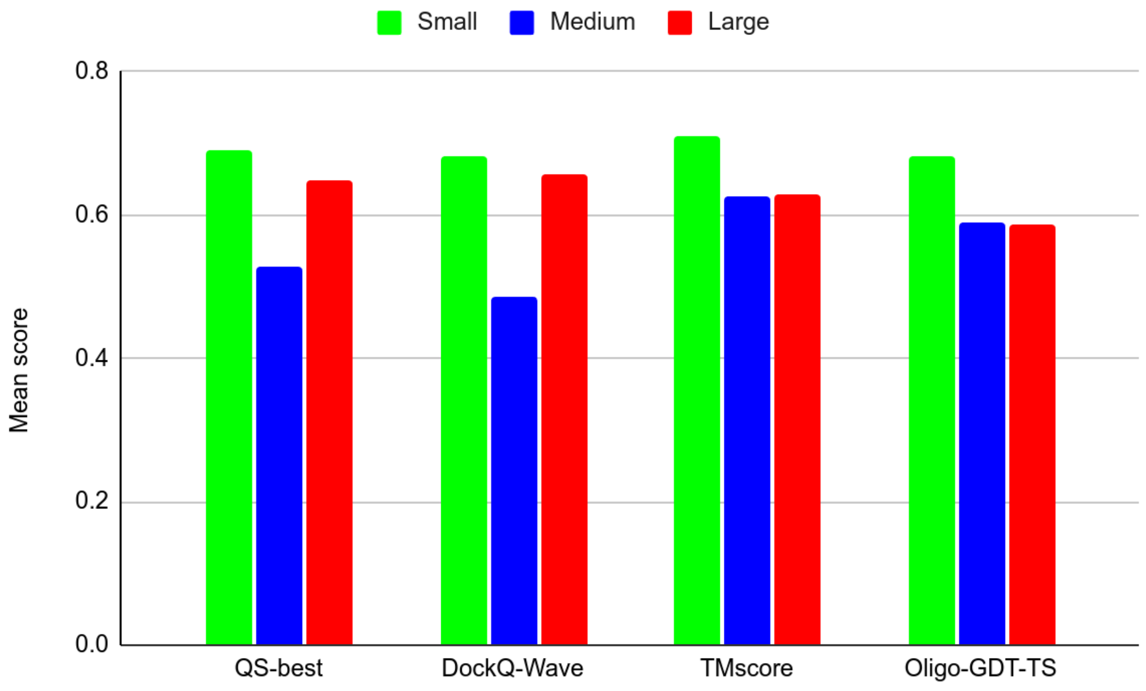

Supplement: gkaf336_Supplemental_File [file gkaf336_supplemental_file.pdf]
